# A *Saccharomyces cerevisiae* model and screen to define the functional consequences of oncogenic histone missense mutations

**DOI:** 10.1093/g3journal/jkac120

**Published:** 2022-05-14

**Authors:** Laramie D Lemon, Sneha Kannan, Kim Wai Mo, Miranda Adams, Haley G Choi, Alexander O D Gulka, Elise S Withers, Hasset T Nurelegne, Valeria Gomez, Reina E Ambrocio, Rhea Tumminkatti, Richard S Lee, Morris Wan, Milo B Fasken, Jennifer M Spangle, Anita H Corbett

**Affiliations:** Department of Biology, Emory University, Atlanta, GA 30322, USA; Department of Biology, Emory University, Atlanta, GA 30322, USA; Department of Biology, Emory University, Atlanta, GA 30322, USA; Department of Biology, Emory University, Atlanta, GA 30322, USA; Department of Radiation Oncology, Emory University, Atlanta, GA 30322, USA; Graduate Program in Cancer Biology, Emory University, Atlanta, GA 30322, USA; Department of Biology, Emory University, Atlanta, GA 30322, USA; Department of Radiation Oncology, Emory University, Atlanta, GA 30322, USA; Department of Biology, Emory University, Atlanta, GA 30322, USA; Graduate Program in Genetics and Molecular Biology, Emory University, Atlanta, GA 30322, USA; Department of Biology, Emory University, Atlanta, GA 30322, USA; Department of Biology, Emory University, Atlanta, GA 30322, USA; Department of Biology, Emory University, Atlanta, GA 30322, USA; Department of Biology, Emory University, Atlanta, GA 30322, USA; Department of Biology, Emory University, Atlanta, GA 30322, USA; Department of Biology, Emory University, Atlanta, GA 30322, USA; Department of Radiation Oncology, Emory University, Atlanta, GA 30322, USA; Department of Biology, Emory University, Atlanta, GA 30322, USA; Department of Biology, Emory University, Atlanta, GA 30322, USA; Department of Radiation Oncology, Emory University, Atlanta, GA 30322, USA; Department of Biology, Emory University, Atlanta, GA 30322, USA

**Keywords:** histone, oncohistone, *Saccharomyces cerevisiae*, gene expression, high copy suppressor

## Abstract

Somatic missense mutations in histone genes turn these essential proteins into oncohistones, which can drive oncogenesis. Understanding how missense mutations alter histone function is challenging in mammals as mutations occur in a single histone gene. For example, described oncohistone mutations predominantly occur in the histone *H3.3* gene, despite the human genome encoding 15 H3 genes. To understand how oncogenic histone missense mutations alter histone function, we leveraged the budding yeast model, which contains only 2 H3 genes, to explore the functional consequences of oncohistones H3K36M, H3G34W, H3G34L, H3G34R, and H3G34V. Analysis of cells that express each of these variants as the sole copy of H3 reveals that H3K36 mutants show different drug sensitivities compared to H3G34 mutants. This finding suggests that changes to proximal amino acids in the H3 N-terminal tail alter distinct biological pathways. We exploited the caffeine-sensitive growth of H3K36-mutant cells to perform a high copy suppressor screen. This screen identified genes linked to histone function and transcriptional regulation, including Esa1, a histone H4/H2A acetyltransferase; Tos4, a forkhead-associated domain-containing gene expression regulator; Pho92, an N6-methyladenosine RNA-binding protein; and Sgv1/Bur1, a cyclin-dependent kinase. We show that the Esa1 lysine acetyltransferase activity is critical for suppression of the caffeine-sensitive growth of H3K36R-mutant cells while the previously characterized binding interactions of Tos4 and Pho92 are not required for suppression. This screen identifies pathways that could be altered by oncohistone mutations and highlights the value of yeast genetics to identify pathways altered by such mutations.

## Introduction

Virtually every somatic cell within a eukaryotic organism contains identical genetic information; however, this identical information produces a plethora of cells with different morphologies and functions. Precise regulation of gene expression enables cells to have specific functions, structures, and biological responses. To enable dynamic responses via gene expression, DNA is packaged with histone proteins to form the basic chromatin unit of the nucleosome, which is a complex containing 147 bp of DNA wrapped around a histone octamer containing 2 copies each of the histones H2A, H2B, H3, and H4. DNA and histone proteins are modified by post-translational modifications (PTMs), which modulate DNA accessibility and regulate gene expression (Strahl and Allis 2000). Histones themselves are key regulators of nucleosome accessibility as a result of their dynamic acetylation, phosphorylation, and methylation. The exact combination, genome-wide localization, and dynamic addition or removal of histone PTMs contribute to the plasticity of gene expression, which is often deregulated in disease.

While the mechanisms by which characterized mutations in histone genes impact gene expression vary, expression of several disease-associated histone missense protein variants leads to chromatin remodeling and aberrant gene expression. To date, mutations in the genes that encode histone H3 have been most extensively characterized (Wan *et al.* 2018; Nacev *et al.* 2019). In particular, data suggest that the H3K27M mutation decreases genome-wide repressive H3K27 methylation via impaired function of the polycomb repressive complex 2 (PRC2) (Lewis *et al.* 2013; Justin *et al.* 2016; Harutyunyan *et al.* 2019). Loss of H3K27 methylation is visible across all genomic elements examined, including promoters, introns, intergenic regions, and 5′- and 3′-untranslated regions (UTRs) (Harutyunyan *et al.* 2019). Tissue characterized by H3K27M mutation also exhibits global DNA hypomethylation, leading to reduced promoter H3K27 methylation. Similarly, the H3K36M mutation is associated with a global reduction in H3K36 di- and tri-methylation, modulating gene expression, and ultimately deregulating biological processes including cellular differentiation (Lu *et al.* 2016). Purified H3K36M-containing nucleosomes inhibit the activity of the H3K36-directed methyltransferases SETD2 and NSD2, and knockdown of these H3K36 methyltransferases phenocopies the effect H3K36M has on the epigenome and gene expression (Yang *et al.* 2016). These data suggest that while known histone mutations deregulate the epigenome and have drastic effects on gene expression, mechanisms by which histone mutations alter cell/biological function likely vary widely and these mutations have the potential to provide insight into specific histone functions.

The human genome encodes more than a dozen of each histone gene, H1, H2A, H2B, H3, and H4, which have arisen evolutionarily at least in part via gene duplication (Braastad *et al.* 2004). All known and characterized disease-associated histone mutations (e.g. H3K27M, H3G34R, H3G34V, and H3K36M) act as dominant mutations, as a mutation in a single copy of one of the 15 encoded human H3 genes is sufficient to impart biological changes that impact cell growth (Nacev *et al.* 2019). Oncohistone mutations that occur within N-terminal histone tails typically function through the deregulation of chromatin modification; in cases such as H3K27M or H3K36M histone variants, these mutations directly impact the ability of H3K27 or H3K36 to support methylation. As described above for H3K27M, the impact on histone PTM occurs both in *cis* and *trans* (Yang *et al.* 2016); similar results are reported for H3K36M-driven cancers (Taylor and Westendorf 2021). These global changes to chromatin have the potential to deregulate transcriptional competence and subsequent gene expression on a large scale, which presents a new obstacle toward therapeutic development—how does one identify a deregulated transcriptional target(s) that is of consequence to cancer development? While recent studies have defined the dopamine receptor DRD2 as a therapeutically actionable gene target deregulated via H3K27M oncohistone expression (Zhang *et al.* 2021), the identification of biologically meaningful cellular pathways and transcriptional changes that oncohistone-driven tumors are acutely dependent upon remains a pressing unmet clinical need.

While genomic alterations that support oncogenic growth have been identified in histone H1 (Yusufova *et al.* 2021) and histone H2B genes (Kang *et al.* 2021; Wan and Chan 2021), most studies have focused on the histone H3 genes (Nacev *et al.* 2019), in which typically 1 mutation is identified amongst the 15 genes, or 30 alleles, encoding H3 in the human genome. Oncogenic H3 mutations usually cluster in either of 2 genes encoding the H3 variant H3.3, which is the most evolutionarily conserved histone H3 variant (Nacev *et al.* 2019). Studies on histones in model organisms including the budding yeast *S. cerevisiae* (Duina *et al.* 2014; Bagert *et al.* 2021) and fission yeast *Schizosaccharomycespombe* (Yadav *et al.* 2017; Lowe *et al.* 2021) can circumvent the complexity of studying the large number of histone gene copies in the human genome and provide insight into how histone mutations impart biologically relevant functional consequences. Such studies are informative as histones are highly conserved across species. Importantly, human and yeast H3 proteins have >97% identity (Hyland *et al.* 2005). While the fission yeast *S. pombe* genome harbors 3 histone H3 genes, the budding yeast *S. cerevisiae* genome contains only 2 histone H3 genes, *HHT1* and *HHT2*, rendering either yeast model system amenable to the study of how the oncohistone changes in histone H3 alter histone function.

Previous studies in *S. pombe* have demonstrated that the oncogenic H3G34 mutations—H3G34V, H3G34W, and H3G34R—function through divergent mechanisms. The H3G34V mutation reduces H3K36 trimethylation (H3K36me3) (Yadav *et al.* 2017), whereas the H3G34R mutation impairs Gcn5-mediated H3K36 acetylation (Lowe *et al.* 2021). Experiments in *S. pombe* also highlight the different mechanisms of action of oncohistone mutations, as H3G34V-mutant yeast are sensitive to DNA-damaging agents, despite having intact homologous recombination-based DNA repair but are not sensitive to replicative stress (Lowe *et al.* 2021). In contrast, H3G34R-mutant yeast are vulnerable to replicative stress but are not competent for homologous recombination DNA repair (Yadav *et al.* 2017). These mutant oncohistones drive differential gene expression changes in yeast, which likely contribute to the contrasting sensitivities of the cells to stress. Such yeast studies highlight the concept that cancers that arise as a result of a specific oncohistone mutation are likely to benefit from different therapeutic modalities.

Similar results in yeast have been observed for various H3K36 amino acid substitutions. Expression of an H3K36R variant, which although structurally similar to the lysine normally present in this position, cannot be post-translationally modified (Meers *et al.* 2017), in *S. cerevisiae* renders yeast acutely sensitive to caffeine (McDaniel *et al.* 2017). Notably, H3K36R-expressing yeast cells exhibit a loss of H3K36me3 and compromised growth in response to caffeine and rapamycin, which both inhibit growth factor and nutrient sensing pathways (McDaniel *et al.* 2017). The H3K36R mutation also alters alternative polyadenylation and pre-mRNA splicing in budding yeast (Sorenson *et al.* 2016; Kaczmarek Michaels *et al.* 2020). The H3K36R missense mutation is not linked to cancer, but it does alter the cellular transcriptional program and impair post-transcriptional processing in a *Drosophila melanogaster* model (Meers *et al.* 2017). In addition, an H3K36A mutation increases antisense transcription in budding yeast (Venkatesh *et al.* 2016). All of these studies demonstrate the utility of employing budding yeast to explore the functional consequences of changing key amino acids within the histone H3 N-terminal tail.

Here, we leverage the budding yeast model system to further elucidate the fundamental biological differences and vulnerabilities amongst established H3G34 and H3K36 oncohistones. Using a high copy suppressor screen approach to identify suppressors of oncohistone-mutant growth phenotypes, we identify several genes linked to histone function, including the histone acetyltransferase, Esa1, the cyclin-dependent kinase, Sgv1/Bur1, a histone deacetylase (HDAC) complex-interacting protein, Tos4, and, finally, a protein that regulates mRNA stability and binds m^6^A RNA, Pho92. Notably, we find that the histone acetyltransferase activity of Esa1 is required for suppression of the caffeine-sensitive growth of H3K36R-mutant cells. Such approaches have the potential to guide novel mechanistic studies of human oncogenic histone mutations and suggest opportunities for therapeutic intervention for patients with tumors characterized by these oncohistones.

## Materials and methods

### Chemicals and media

All chemicals were obtained from Sigma-Aldrich (St Louis, MO, USA), United States Biological (Swampscott, MA, USA), or Fisher Scientific (Pittsburgh, PA, USA) unless otherwise noted. All media were prepared by standard procedures (Adams *et al.* 1997).

### *Saccharomyces cerevisiae* strains and plasmids

All DNA manipulations were performed according to standard procedures (Sambrook *et al.* 1989). *Saccharomycescerevisiae* strains and plasmids used in this study are listed in Supplementary Table 1. The PCR- and homologous recombination-based system for generating targeted mutations in histone genes in budding yeast cells has been described (Duina and Turkal 2017). Strains to model oncohistones—*hht2-K36R* (ACY2816), *hht2-K36M* (ACY2830), *hht2-G34W* (ACY2823), *hht2-G34L* (ACY2831), *hht2-G34R* (ACY2838), and *hht2-G34V* (ACY2841), which harbor mutations at the codons encoding the 36th or 34th histone H3 residue at the endogenous *HHT2* gene—were generated using the parental *hht2Δ::URA3* strain (yAAD165) and the strategy detailed previously (Johnson *et al.* 2015; Duina and Turkal 2017). The endogenous *HHT1* gene in these oncohistone model strains was subsequently deleted and replaced via homologous recombination with a *kan*MX marker cassette to generate *hht2-K36R/M hht1Δ* (ACY2821, ACY2822) and *hht2-G34W/L/R/V hht1Δ* (ACY2825, ACY2833, ACY2840, ACY2846) strains. The *hht1Δ* (ACY2818) and *set2Δ* strains (ACY2851) were generated by deletion and replacement of the *HHT1* and *SET2* gene, respectively, via homologous recombination with a *kan*MX marker cassette. The YEp352 plasmids containing cloned *S. cerevisiae* genes identified in the high copy suppressor screen—*HHF2* (pAC4199), *HHT2* (pAC4201), *ESA1* (pAC4190), *TOS4* (pAC4196), *PHO92* (pAC4193), *SGV1* (pAC4187), and *HHT1* (pAC4200)—were generated by PCR amplification of each gene (5′ sequence/promoter, CDS, 3′ UTR) from wildtype (WT) BY4741 genomic DNA with gene-specific oligonucleotides (Integrated DNA Technologies) and conventional cloning via *Sal*I/*Sac*I or *Xho*I/*Sph*I (*TOS4*) into YEp352. All enzymes for PCR and cloning were obtained from New England BioLabs. The YEp352 plasmids containing *esa1/tos4/pho92/sgv1* variants with missense mutations in the Esa1 catalytic domain—*esa1-C304S* (pAC4191) and *esa1-E338Q* (pAC4192), the Tos4 Forkhead-associated (FHA) domain—*tos4-N122A-N161A* (pAC4205), the Pho92 YTH domain—*pho92-W177A* (pAC4194) and *pho92-W231A* (pAC4195), and the Sgv1 catalytic domain—*sgv1-E107Q* (pAC4188), *sgv1-D213A* (pAC4189), *SUP3-E107Q* (pAC4212), and *SUP3-D213A* (pAC4213) were created using oligonucleotides containing the desired mutations (Integrated DNA Technologies), plasmid template—*ESA1* (pAC4190), *TOS4* (pAC4196), *PHO92* (pAC4193), *SGV1* (pAC4187), or *SUP3* (pAC4132), and the QuikChange II Site-Directed Mutagenesis Kit (Agilent). The YEp352 plasmids containing *sgv1Δ2 + 8aa* (pAC4208), *SGV1-Myc* (pAC4209), *sgv1-E107Q-Myc* (pAC4210), and *sgv1-D213A-Myc* (pAC4211) were generated by PCR amplification of *SGV1* 5′ sequence/CDS and 3′ UTR products from *SGV1* plasmid template—*SGV1* (pAC4187), *sgv1-E107Q* (pAC4188), or *sgv1-D213A* (pAC4189) using oligonucleotides containing desired changes (Δ2 + 8aa; C-terminal Myc tag) and overlapping ends (Integrated DNA Technologies), and cloning into YEp352 linearized with *Eco*RI/*Hind*III using the NEBuilder Hifi DNA assembly cloning kit (New England BioLabs). All clones generated were sequenced to ensure that all cloned WT *S. cerevisiae* genes contained no mutations and all gene variants contained only the desired mutations.

### High-copy suppressor screen

High-copy suppressors of the caffeine**-**sensitive growth of *hht2-K36R hht1Δ* cells (ACY2821) or *hht2-K36M hht1Δ* cells (ACY2822) were identified by transforming these cells with a YEp352-based high-copy *S. cerevisiae* genomic library (Zhang *et al.* 1998) which was created by performing a *Sau*3AI partial digest of genomic *S. cerevisiae* DNA and cloning into the *Bam*HI site of the 2 µm plasmid YEp352 (Hill *et al.* 1986). Transformants were grown on synthetic medium lacking uracil in the presence of 15 mM caffeine at 30°C for 5–10 days to select for those plasmids able to complement the caffeine**-**sensitive growth phenotype. Suppression by each plasmid (plasmid linkage) was confirmed by rescuing each suppressor plasmid and then retransforming them into the original H3K36 mutant to test for suppression of the slow growth on plates containing 15 mM caffeine.

### *Saccharomyces cerevisiae* growth assays

To examine the growth of oncohistone model strains, WT (yAAD1253), *hht2-K36R/M Δhht1* (ACY2821; ACY2822), *hht2-G34W/L/R/V hht1Δ* (ACY2825; ACY2833; ACY2840; ACY2846), and *hht1Δ* (ACY2818) strains were grown overnight at 30°C to saturation in 2 ml YEPD (yeast extract, peptone, dextrose) media. Cells were normalized to OD_600_ = 5, serially diluted in 10-fold dilutions, spotted on control YEPD media plates or YEPD media plates containing 15 mM caffeine or 3% formamide, and grown at 30°C and 18°C for 2–5 days. To test the effect of the genomic suppressor plasmids on the growth of oncohistone model strains and *set2Δ* cells in the presence of caffeine, WT (yAAD1253) cells transformed with YEp352 plasmid and *hht2-K36M hht1Δ* (ACY2822), *hht2-K36R hht1Δ* (ACY2821), and *set2Δ* (ACY2851) cells transformed with Vector (YEp352), *SUP3* (pAC4132), *SUP54* (pAC4145), *SUP67* (pAC4149), *SUP68* (pAC4150), or *SUP99* (pAC4160) plasmid were grown overnight at 30°C to saturation in 2 ml Ura− media containing 2% glucose. Cells were normalized by OD_600_ and serially diluted as described previously, spotted onto control YEPD media plates or YEPD media plates containing 15 mM caffeine, and grown at 30°C for 2–5 days. For some experiments, cells were also spotted onto control Ura− plates or Ura− plates containing 15 mM caffeine. To test the effect of cloned suppressor genes and gene variants on the growth of *hht2-K36R hht1Δ* cells in the presence of caffeine, WT (yAAD1253) cells containing Vector (YEp352) and *hht2-K36R hht1Δ* (ACY2821) cells containing *HHF2* (pAC4199), *HHT2* (pAC4201), *HHT1* (pAC4200), *ESA1* (pAC4190), *esa1-C304S* (pAC4191), *esa1-E338Q* (pAC4192), *TOS4* (pAC4196), *tos4-R122A-N161A* (pAC4205), *PHO92* (pAC4193), *pho92-W177A* (pAC4194), *pho92-W231A* (pAC4195), *SGV1* (pAC4187), *sgv1-Δ2 + 8aa* (pAC4208), *SGV1-Myc* (pAC4209), *sgv1-E107Q-Myc* (pAC4210), *sgv1-D213A-Myc* (pAC4211), *SUP3-E107Q* (pAC4212), or *SUP3-D213A* (pAC4213) plasmid were grown overnight at 30°C to saturation in 2 ml Ura− media containing 2% glucose. As controls and for comparison, *hht2-K36R hht1Δ* (ACY2821) cells containing genomic suppressor plasmids were similarly grown. Cells were normalized by OD_600_ and serially diluted as described previously, spotted onto control YEPD plates or YEPD media plates containing 15 mM caffeine, and grown at 30°C for 2–5 days. For some experiments, cells were also spotted onto control Ura− plates or Ura− plates containing 15 mM caffeine. For liquid growth assays, the indicated genotypes cells were grown in YEPD to mid-log phase and diluted into fresh media. The OD_600_ was recorded every 20 min in an Epoch2 microplate reader (BioTek) to determine doubling time. For the results shown, each sample was performed in 3 independent biological replicates with 3 technical replicates for each biological sample.

### Histone immunoblotting

To analyze histone H3K36me3 levels in oncohistone model strains, WT (yAAD1253), *hht2-K36R/M Δhht1* (ACY2821; ACY2822), *hht2-G34W/L/R/V hht1Δ* (ACY2825; ACY2833; ACY2840; ACY2846), and *set2Δ* (ACY2851) strains were grown overnight at 30°C to saturation in 5 ml YEPD (yeast extract, peptone, dextrose) media. Cells were diluted in 50 ml YEPD to a starting OD_600_ = 0.1 and grown at 30°C to a final OD_600_ = 1.0. Cells were pelleted by centrifugation at 1,962 × *g* in 50 ml tubes and transferred to 2 ml screwcap tubes and pelleted by centrifugation at 16,200 × *g*. Pelleted cells were resuspended in 1 ml Lysis Buffer (10 mM Tris HCl, pH 8.0; 300 mM NaCl; 10% Glycerol; 0.1% IGEPAL CA-630) supplemented with protease inhibitors [0.5 mM PMSF; Pierce Protease Inhibitors (Thermo Fisher Scientific)]. After addition of 500 µl acid washed glass beads, cells were disrupted in a Mini Bead Beater 16 Cell Disrupter (Biospec) for 3 × 30 s at 25°C with 1 min on ice between repetitions. Cell debris was pelleted by centrifugation at 2,400 × *g* for 2 min at 4°C and protein lysate supernatant was transferred to fresh microfuge tube and clarified by centrifugation at 16,200 × *g* for 15 min at 4°C. The protein lysate was then transferred to a fresh tube. Protein lysate concentration was determined by Pierce BCA Protein Assay Kit (Life Technologies). Protein lysate samples (60 µg) in reducing sample buffer (50 mM Tris HCl, pH 6.8; 100 mM DTT; 2% SDS; 0.1% bromophenol blue; 10% glycerol) were resolved on 4–20% Criterion TGX Stain-Free precast polyacrylamide gels (Bio-Rad). Protein lysate samples were transferred to nitrocellulose membranes (Bio-Rad) in Dunn carbonate buffer (10 mM NaHCO_3_, 3 mM Na_2_CO_3_, pH 9.9, 20% methanol) at 22 V for 90 min at room temperature. Histone H3K36me3 was detected with anti-H3K36me3 rabbit polyclonal antibody (ab9050; 1:1000; Abcam) and total histone H3 was detected with anti-H3 rabbit polyclonal antibody (ab1791; 1:5000; Abcam). Primary H3 rabbit antibodies were detected with secondary peroxidase-conjugated goat anti-rabbit IgG (111-035-003; 1:3000; Jackson ImmunoResearch Labs, Inc.), ECL reagent, and ChemiDoc MP Imaging System (Bio-Rad).

### Quantitation of histone immunoblotting

The protein band intensities from immunoblots were quantitated using Image Lab software (Bio-Rad) and mean fold changes in protein levels were calculated in Microsoft Excel (Microsoft Corporation). The mean fold changes in histone H3K36me3 levels in oncohistone-mutant cells relative to the WT control was calculated from 2 immunoblots. H3K36me3 band intensity was first normalized to total histone H3 band intensity and then normalized to H3K36me3 intensity in WT cells. The mean fold changes in H3K36me3 levels in oncohistone-mutant cells relative to the WT control were graphed in GraphPad Prism 8 (GraphPad Software, LLC) with standard error of the mean error bars.

## Results

### Missense mutations that model changes present in histone H3 confer different growth phenotypes

**Fig. 1.**
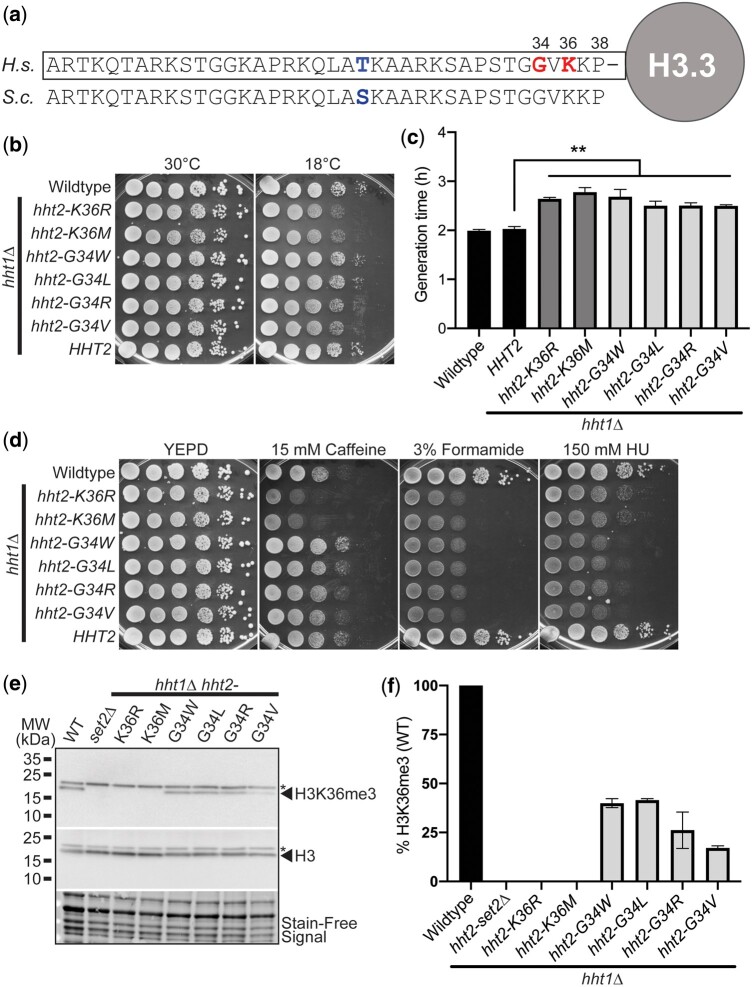
H3K36 and H3G34 oncohistone missense mutations within the conserved N-terminal tail of histone H3 cause diverse growth phenotypes in budding yeast. a) The N-terminal tails of histone proteins are highly conserved as illustrated in the alignment shown for human (*H. sapiens*, *H.s.*) histone H3.3 and *S. cerevisiae* (*S.c.*) Hht1/Hht2. The positions of K36 and G34, which are the residues altered in the oncohistones modeled here, are shown in bold on right in red and the single conservative amino acid change from threonine (T) in human to serine (S) in budding yeast at position 22 is shown in bold towards center in blue. b) The H3K36 histone-mutant cells, but not H3G34-mutant cells, show cold-sensitive growth. The H3K36R/M-mutant cells (*hht2-K36R/M hht1Δ*) show impaired growth at 18°C compared to WT cells. The H3K36 and H3G34-mutant cells express each histone variant as the sole copy of histone H3. WT, H3K36-mutant cells (*hht2-K36R/M hht1Δ*), H3G34-mutant cells (*hht2-G34W/L/R/V hht1Δ*), and *HHT2 hht1Δ* cells were serially diluted and spotted on YEPD media plates and grown at the permissive temperature of 30°C and cold temperature of 18°C. c) A growth curve was employed to analyze growth rate (Supplementary Fig. 1). Doubling times were obtained for control WT and *HHT2 hht1Δ* cells (black), H3K36-mutant cells (*hht2-K36R/M hht1Δ*) (gray), and H3G34-mutant cells (*hht2-G34W/L/R/V hht1Δ*) (light gray). The doubling time was increased for each of the H3 mutants analyzed compared to either control. Statistical significance (**P < 0.001) was determined by using a 1-tailed Student’s *t*-test. d) The H3K36 histone-mutant cells show caffeine-sensitive growth, whereas H3G34-mutant cells show HU-sensitive growth. The H3K36R/M-mutant cells (*hht2-K36R/M hht1Δ*) show impaired growth on caffeine plates, whereas the H3G34-mutant cells (*hht2-G34W/L/R/V hht1Δ*) show impaired growth on HU plates compared to WT cells. All H3K36- and H3G34-mutant cells analyzed show severely impaired growth on formamide plates. WT, H3K36-mutant cells (*hht2-K36R/M hht1Δ*), H3G34-mutant cells (*hht2-G34W/L/R/V hht1Δ*), and *HHT2 hht1Δ* cells were serially diluted and spotted on a control YEPD media plate and YEPD plates containing 15 mM caffeine, 3% formamide, or 150 mM HU. and grown at 30°C. e and f) Immunoblotting was performed to analyze levels of H3K36 trimethylation (H3K36me3) in budding yeast oncohistone models. WT, control *set2Δ*, H3K36-mutant cells (*hht2-K36R/M hht1Δ*), and H3G34-mutant cells (*hht2-G34W/L/R/V hht1Δ*) were grown and analyzed as described in Materials and methods to detect H3K36me3 and total H3. Stain-free signal is included as a total loading control. e) A representative immunoblot demonstrating loss of H3K36me3 in the control *set2Δ* and H3K36-mutant cells (*hht2-K36R/M hht1Δ*) with a decrease in H3K36me3 in the H3G34-mutant cells. A nonspecific band detected by the antibody is indicated by the asterisk. Molecular weight markers are indicated to the left. f) A bar graph presents the results from 2 independent immunoblot experiments as shown in (e). The level of H3K36me3 was normalized to stain-free signal and total H3 levels. The level of H3K36me3 detected in control WT cells was set to 100% and all other samples were calculated relative to this level of H3K36me3.

The N-terminal tails of the histone H3 protein are evolutionarily conserved with only a single conservative amino acid substitution (T->S) within the first 38 amino acids of the budding yeast compared to the human protein (Fig. 1a). For this reason, the budding yeast system is valuable to model missense mutations that convert histone proteins into oncohistones. We focused on a set of missense mutations that alter K36 (K36M) or neighboring G34 (G34W, G34L, G34R, G34V), which are altered in various types of cancers, and an additional missense mutation that alters K36 and is associated with post-transcriptional regulation (McDaniel *et al.* 2017) (K36R).

We created *S. cerevisiae* H3-mutant models where each histone variant is expressed as the sole copy of histone H3 and then assessed cell growth using a serial dilution assay. As shown in Fig. 1b, in an end point solid media growth assay, all histone mutants show growth comparable to either control WT cells or control cells lacking the *HHT1* gene (*HHT2 hht1Δ*), which is absent in the oncohistone-mutant models that contain *HHT2* as the sole histone H3 gene. At cold temperature (18°C), both the H3K36R and H3K36M-mutant cells show a modest growth defect (Fig. 1b) with no change in growth detected for any of the H3G34 variants. To extend this analysis, we performed a liquid growth assay, which captures changes in the rate of growth. At the permissive temperature of 30°C, differences in the growth rate for each of the oncohistone mutants relative to WT control cells are illustrated by increased doubling time (Fig. 1c) and slower growth rate (Supplementary Fig. 1). These data demonstrate that these oncohistone mutants exhibit changes in cell growth.

To explore whether the oncohistone models exhibit other changes, we then tested for growth defects when cells are grown on media containing chemicals that disrupt different cellular pathways (Fig. 1d). Caffeine impairs cellular stress response/TOR signaling (Kuranda *et al.* 2006); formamide alters RNA metabolism (Hoyos-Manchado *et al.* 2017), and hydroxyurea (HU) impairs DNA synthesis (Slater 1973). Results of this analysis reveal that amino acid substitutions at H3K36 cause sensitivity to growth on media containing caffeine, which is consistent with previous results showing that loss of the H3K36 methyltransferase Set2 (Strahl *et al.* 2002) and expression of the H3K36R variant confer sensitivity to caffeine (McDaniel *et al.* 2017). All of the histone H3 mutants are sensitive to formamide, while the H3G34 mutants are more sensitive to HU than the H3K36 mutants.

Analysis of H3K36me3 in the oncohistone-mutant cells reveals that H3K36R and H3K36M mutants show no detectable H3K36me3, as expected and similar to control *set2Δ* cells, whereas H3G34 mutants show decreased H3K36me3 relative to WT cells (Fig. 1, e and f). Notably, amongst the H3G34 mutants, H3G34V shows the greatest decrease in H3K36me3. These results suggest that the drug-sensitive growth of the oncohistone mutants is linked to an altered epigenome. In addition, these data reveal that different changes to residues within the N-terminal tail of histone H3 confer distinct growth defects.

### A high copy suppressor screen to identify functional links to missense mutations present in oncohistones

**Fig. 2.**
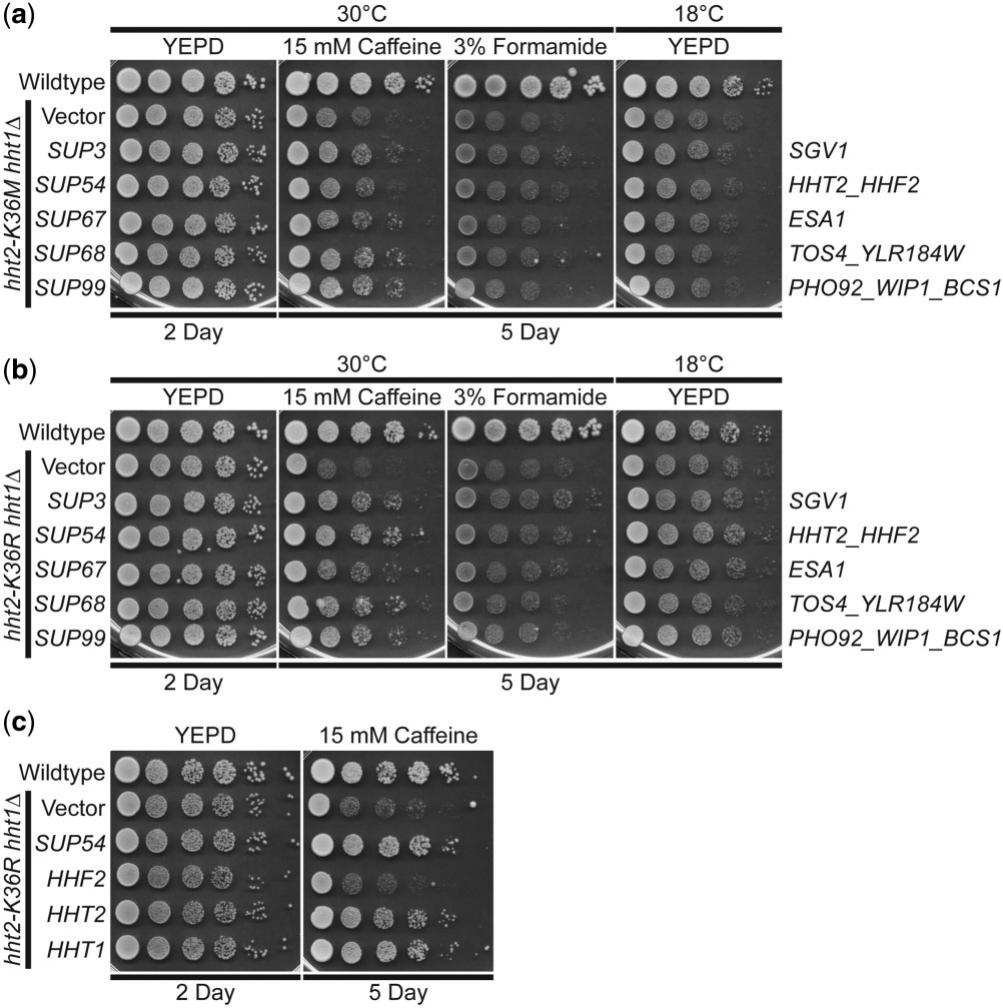
A high copy suppressor screen for suppression of the caffeine-sensitive growth of H3K36 histone-mutant cells identifies 5 suppressors. As described in the Materials and methods, a high copy suppressor screen was performed to identify genes that suppress the caffeine-sensitive growth of H3K36M- and/or H3K36R-mutant cells. The 5 genomic suppressor (*SUP*) plasmids identified from the screen suppress the caffeine-sensitive growth of a) H3K36M and b) H3K36R histone-mutant cells. The H3K36M/R-mutant cells (*hht2-K36M/R hht1Δ*) containing *SUP3/54/67/68/99* suppressor plasmids show improved growth on caffeine plates compared to cells containing vector alone. The H3K36M/R cells containing suppressor plasmids do not show any effect on growth at 18°C and little effect on formamide plates; however, cells containing the *SUP3* suppressor show slightly improved growth compared to cells containing vector alone on formamide plates. Sequencing of the genomic suppressor plasmids revealed the identity of the gene(s) encoded on the clones: *SUP3* (*SGV1*), *SUP54* (*HHT2, HHF2*), *SUP67* (*ESA1*), *SUP68* (*TOS4, YLR184W*), and *SUP99* (*PHO92, WIP1, BCS1*)—indicated to right. WT cells transformed with vector and H3K36M/R-mutant cells (*hht2-K36M/R hht2Δ*) transformed with vector, *SUP3*, *SUP54*, *SUP67*, *SUP68*, or *SUP99* plasmid were serially diluted and spotted onto YEPD plates and YEPD plates containing 15 mM caffeine or 3% formamide and grown at indicated temperatures. c) The *HHT2* histone H3 gene, but not the *HHF2* histone H4 gene, suppresses the caffeine-sensitive growth of H3K36R histone-mutant cells. The H3K36R-mutant cells (*hht2-K36R hht1Δ*) containing an *HHT2* plasmid show improved growth compared to cells with vector alone on a caffeine plate, similar to cells containing the *SUP54* suppressor, which harbors both *HHT2* and *HHF2*. The H3K36R cells containing an *HHF2* plasmid do not show improved growth on a caffeine plate. The H3K36R cells with an *HHT1* plasmid, containing the other *S. cerevisiae* histone H3 gene, show improved growth on a caffeine plate. WT cells transformed with vector and H3K36R-mutant cells (*hht2-K36R hht2Δ*) transformed with vector, *SUP54*, *HHF2*, *HHT2*, or *HHT1* plasmid were serially diluted and spotted onto a control YEPD plate and YEPD plate containing 15 mM caffeine and grown at 30°C.

To explore the molecular basis of the growth defects conferred by missense mutations in the histone H3 gene, we performed a high copy suppressor screen. As described in Materials and methods, either H3K36M- or H3K36R-mutant cells were transformed with a high copy genomic library (Zhang *et al.* 1998) and plated on media containing 15 mM caffeine. Suppressors that enhanced growth of these mutants in the presence of caffeine were isolated and selected for further analysis. Figure 2a shows results for a set of suppressor clones (*SUP*) identified in the screen that suppress the caffeine-sensitive growth of the H3K36M oncohistone model. While the screen was not saturated, we did identify several clones multiple times. We validated each suppressor by plasmid rescue and retransformation.

As expected in a suppressor screen, we identified a suppressor that contains one of the 2 budding yeast H3 genes, *HHT2* (Fig. 2a). Beyond *HHT2*, which validates the screen, we focused our analysis on 4 suppressor clones that illustrate the power of this approach to define the biological pathways altered in oncohistone model cells. These suppressors encode the cyclin-dependent protein kinase, Sgv1/Bur1 (Irie *et al.* 1991; Prelich and Winston 1993), the catalytic subunit of the NuA4 histone acetyltransferase complex, Esa1 (Clarke *et al.* 1999), the gene expression regulator, Tos4 (Horak *et al.* 2002; Cooke *et al.* 2021), which contains a FHA domain that interacts with Rpd3L and Set3 HDAC complexes (Bastos de Oliveira *et al.* 2012), and a post-transcriptional regulator of phosphate and glucose metabolism, Pho92 (Kang *et al.* 2014) (Fig. 2a).

To begin to assess whether the suppressors identified are linked to PTM of H3K36, we tested whether the suppressors identified can also suppress a yeast mutant that expresses the conservative H3K36R variant as the sole copy of histone H3. As shown in Fig. 2b, each of the suppressors identified also suppresses the caffeine-sensitive growth of H3K36R cells. We also confirmed that each of these suppressors can suppress the H3K36R caffeine-sensitive growth on plates lacking uracil (Supplementary Fig. 2), a condition which has been used in previous studies that analyzed H3K36R cells (McDaniel *et al.* 2017). Thus, the suppressors identified for further analysis suppress the growth phenotypes of both H3K36M- and H3K36R-mutant cells.

As a first step to validate the screen and the suppressors identified, we focused on the suppressor clone containing the histone H3 gene *HHT2* (*SUP54*). This genomic clone contains one of the 2 budding yeast histone H3 genes (*HHT2*) and one of the 2 budding yeast histone H4 genes (*HHF2*). We subcloned the *HHT2* and *HHF2* genes and tested them independently for suppression of the caffeine-sensitive growth of the H3K36R cells (Fig. 2c and Supplementary Fig. 2a). This analysis reveals that *HHT2* rescues the growth defect of these cells, while the *HHF2* clone confers no rescue as cells show growth comparable to the Vector alone control. We also confirmed that, as expected, the other budding yeast histone H3 gene, *HHT1*, rescues the caffeine-sensitive growth of H3K36R-mutant cells (Fig. 2c).

**Fig. 3.**
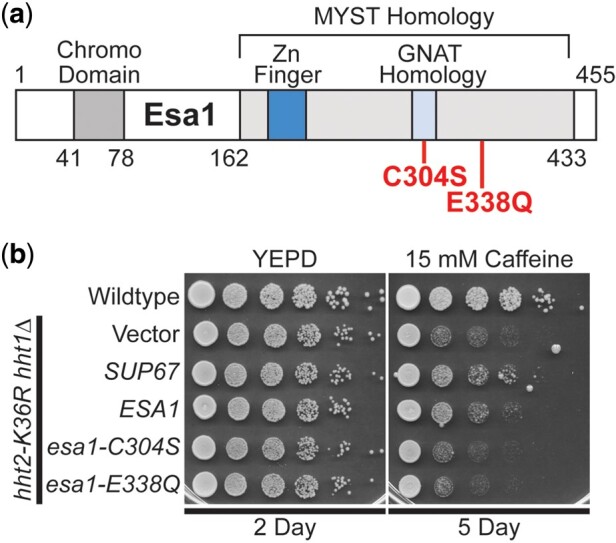
The lysine acetyltransferase *ESA1* suppresses the caffeine-sensitive growth of H3K36R histone-mutant cells and requires the catalytic activity of Esa1 for suppression. *SUP67* includes a single intact gene *ESA1*, which encodes a histone H4/H2A lysine acetyl transferase of the MYST (Moz, YBF2, Sas2, Tip) family. a) The domain structure of Esa1 is shown. The MYST homology domain contains both a Zinc Finger (Zn Finger) domain and a Gcn5-related *N*-acetyltransferases (GNAT) Homology domain. Esa1 also contains an N-terminal chromo domain. The amino acid changes created to impair the lysine acetyltransferase activity of Esa1, C304S and E338Q, which are based on previous work (Decker *et al.* 2008) and located in the MYST homology domain are shown below the diagram in bolded text. b) *ESA1* suppresses the caffeine-sensitive growth of H3K36R-mutant cells similar to the high copy suppressor, *SUP67*, but catalytically inactive mutants of Esa1 do not suppress. The H3K36R cells (*hht2-K36R hht1Δ*) containing an *ESA1* plasmid show improved growth compared to cells with vector alone on a caffeine plate, similar to cells containing the *SUP67* suppressor, but cells containing the catalytically inactive mutant *esa1-C304S* or *esa1-E338Q* plasmid do not show improved growth. WT cells transformed with vector and H3K36R-mutant cells (*hht2-K36R hht2Δ*) transformed with vector, *SUP67*, *ESA1*, *esa1-C304S*, or *esa1-E338Q* plasmid were serially diluted and spotted onto a control YEPD plate and YEPD plate containing 15 mM caffeine and grown at 30°C for indicated days.

The *S. cerevisiae ESA1* gene encodes an essential, evolutionarily conserved lysine acetyltransferase that acetylates lysine residues within the N-terminal tail of histone H4 as well as histone H2A (Smith *et al.* 1998; Clarke *et al.* 1999). The human orthologue of Esa1 is the KAT5/TIP60 protein (Doyon *et al.* 2004). As illustrated in the domain structure shown in Fig. 3a, Esa1 is a member of the MYST (MOZ[KAT6A], YBF2/Sas3, Sas2, TIP60[KAT5]) family of lysine acetyl transferases (Yan *et al.* 2000; Lafon *et al.* 2007). Esa1 also contains an N-terminal chromo domain (Eissenberg 2012). These same functional domains are conserved within the human KAT5/TIP60 protein (Doyon *et al.* 2004).

We confirmed that overexpression of *ESA1*, the only intact gene present in the *SUP67* suppressor, can suppress the caffeine-sensitive growth of H3K36R-mutant cells (Fig. 3b and Supplementary Fig. 2b). To assess whether the lysine acetyltransferase function of Esa1 is critical for this growth suppression, we took advantage of 2 previously characterized catalytic mutants (Fig. 3a), *esa1-C304S* and *esa1-E338Q* (Yan *et al.* 2000; Decker *et al.* 2008). Each of these amino acid substitutions eliminates the acetyltransferase activity of Esa1 without a significant impact on steady-state protein level (Decker *et al.* 2008). As shown in Fig. 3b and Supplementary Fig. 2b, neither of these catalytic mutants of Esa1 can suppress the growth of H3K36R-mutant cells. Thus, the acetyltransferase activity of Esa1 is required to suppress the growth defects associated with H3K36 mutants.

**Fig. 4.**
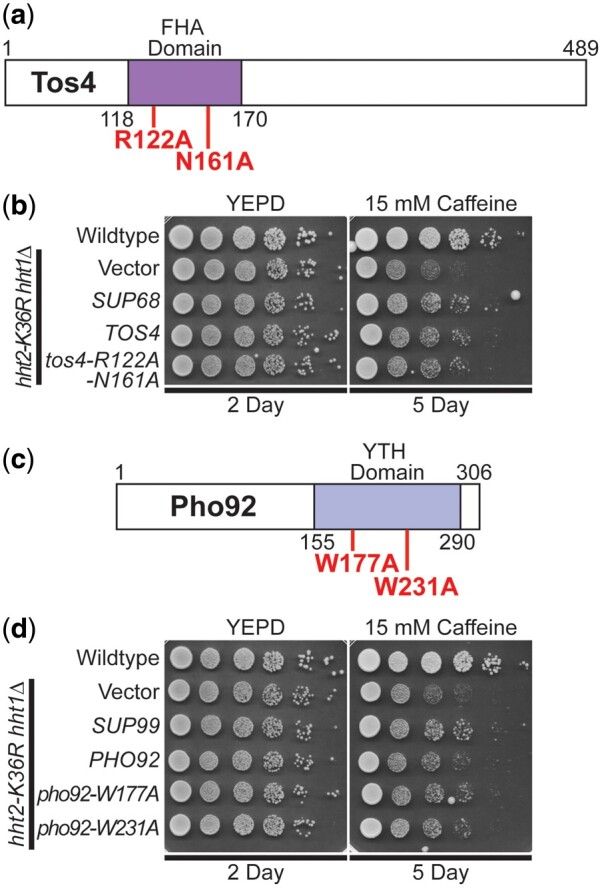
The gene expression regulator *TOS4* and m^6^A RNA-binding protein *PHO92* suppress the caffeine-sensitive growth of H3K36R histone-mutant cells, but Tos4 interaction with HDAC complexes and Pho92 m^6^A RNA binding are not required for suppression. a) The Tos4 protein is a gene expression regulator that contains a forkhead-associated (FHA) domain, which is a phosphopeptide recognition domain found in many regulatory proteins. The amino acids changes generated to disrupt the interaction with the HDAC complexes, Rpd3L and Set3 (Bastos de Oliveira *et al.* 2012; Cooke *et al.* 2021), R122A and N161A, which are located in the FHA domain, are shown below the diagram in bolded text. b) *TOS4* suppresses the caffeine-sensitive growth of H3K36R-mutant cells to the same extent as the high copy suppressor, *SUP68*, and a FHA domain double mutant of Tos4 that disrupts interaction with Rpd3L and Set3 HDACs remains competent to suppress the cells. The H3K36R cells (*hht2-K36R hht1Δ*) containing a *TOS4* plasmid show improved growth compared to cells with vector alone on a caffeine plate, similar to cells containing the *SUP68* suppressor, which contains uncharacterized open reading frame, *YLR184W*, in addition to *TOS4*. The H3K36R cells containing an FHA domain double mutant of Tos4, *tos4-R122A-N161A*, show improved growth compared to cells with vector alone on a caffeine plate, similar to cells containing *TOS4*. c) The Pho92 protein contains a YT521-B homology (YTH) domain, which is an evolutionarily conserved m^6^A-dependent RNA-binding domain (Xu *et al.* 2015). The amino acid changes made to impair the binding of Pho92 to m^6^A RNA, W177A and W231A, which alter key tryptophan residues in the m^6^A binding pocket (Xu *et al.* 2015) located in the YTH domain, are shown belowthe diagram in bolded text. d) *PHO92* suppresses the caffeine-sensitive growth of H3K36R-mutant cells similar to the high copy suppressor, *SUP99*, but the m^6^A RNA-binding function of Pho92 is not required for suppression. The H3K36R cells (*hht2-K36R hht1Δ*) containing a *PHO92* plasmid show improved growth compared to cells with vector alone on a caffeine plate, similar to cells containing the *SUP99* suppressor, which contains *WIP1* and *BCS1* genes in addition to *PHO92*. The H3K36R cells containing the m^6^A RNA-binding mutant *pho92-W177A* or *pho92-W231A* plasmid show improved growth compared to cells with vector alone on a caffeine plate, similar to cells containing *PHO92*. WT cells transformed with vector and H3K36R-mutant cells (*hht2-K36R hht2Δ*) transformed with vector, *SUP68*, *TOS4*, *tos4-R122A-N161A*, *SUP99*, *PHO92*, *pho92-W177A*, or *pho92-W231A* plasmid were serially diluted and spotted onto a control YEPD plate and YEPD plate containing 15 mM caffeine and grown at 30°C for indicated days.

The Tos4 protein is a gene expression regulator that plays a role in gene expression homeostasis (Horak *et al.* 2002; Cooke *et al.* 2021). As depicted in Fig. 4a, Tos4 contains a FHA domain, which mediates interactions with HDAC complexes, Rpd3L and Set3 (Bastos de Oliveira *et al.* 2012). We cloned *TOS4*, as the *SUP68* suppressor clone slightly truncates the Tos4 open reading frame and also contains an uncharacterized gene *YLR184W.* As shown in Fig. 4b, overexpression of *TOS4* suppresses the caffeine-sensitive growth of H3K36R-mutant cells. To test whether a functional FHA domain is required for Tos4-mediated growth suppression, we exploited amino acid substitutions R122A and N161A in the Tos4 FHA domain that disrupt Tos4 interaction with HDAC complexes, Rpd3L and Set3 (Bastos de Oliveira *et al.* 2012). We created a Tos4 variant that contains both these amino acid substitutions (Fig. 4a); however, this double *tos4-R122A-N161A* mutant still robustly suppresses growth defects of H3K36R-mutant cells, suggesting that a HDAC independent, alternative function of Tos4 mediates this suppression (Fig. 4b and Supplementary Fig. 2c).

The Pho92 protein is a member of an evolutionarily conserved family of proteins that contains a C-terminal YTH domain (Kang *et al.* 2014) (Fig. 4c). The YTH domain can recognize and bind m^6^A-containing RNA (Shi *et al.* 2021), serving as the primary “reader” of this post-transcriptional modification of RNA. The high copy suppressor containing *PHO92* (*SUP99*) also contained 2 other genes, *WIP1* and *BCS1*, so we cloned the *PHO92* gene and found that *PHO92* is indeed a high copy suppressor of the caffeine-sensitive growth of H3K36R-mutant cells (Fig. 4d and Supplementary Fig. 2d). To determine whether YTH-mediated interaction with m^6^A-containing RNA is required for this suppression, we altered conserved tryptophan residues that are critical to form the binding pocket for m^6^A (Theler *et al.* 2014; Xu *et al.* 2015), creating *pho92-W177A* and *pho92-W231A* (Fig. 4c). As shown in Fig. 4d and Supplementary Fig. 2d, both of these *pho92* mutants still suppress the caffeine-sensitive growth of the H3K36R cells, strongly suggesting that the YTH domain is not required for this suppression.

**Fig. 5.**
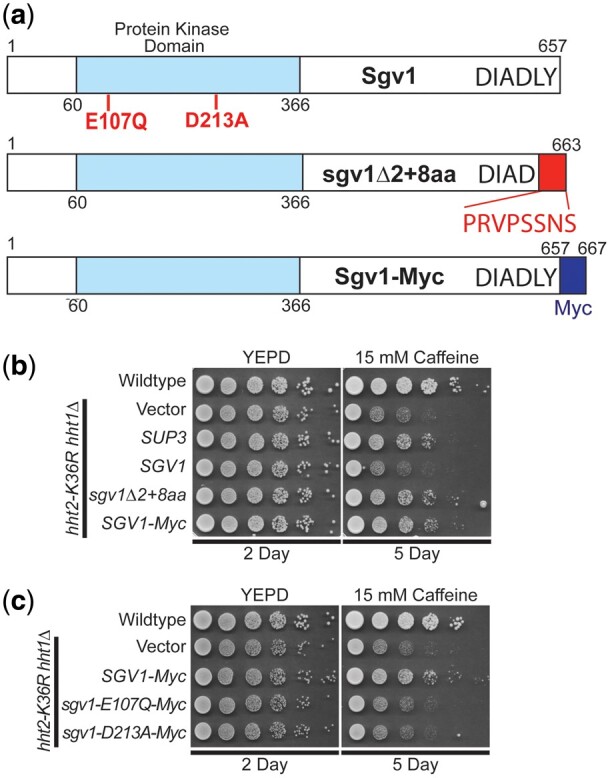
WT *SGV1* does not suppress the caffeine-sensitive growth of H3K36 histone mutants, but Sgv1 with a C-terminal extension can suppress. a) The Sgv1/Bur1 kinase is a cyclin-dependent kinase that contains a protein kinase domain with homology to human CDK9 (Malumbres 2014). The amino acid substitutions generated to inactivate the catalytic function of Sgv1/Bur1 (Keogh *et al.* 2003), E107Q and D213A, which are located in the protein kinase domain, are shown below the diagram in bolded text. The genomic suppressor clone *SUP3* identified in the high copy suppressor screen encodes a slightly truncated Sgv1 protein, which lacks the last 2 amino acids (LY) and translates into the multiple cloning site of the vector (YEp352) to add an additional 8 amino acids (PRVPSSNS), which are shown below the diagram at the C-terminus on the domain structure shown and labeled sgv1Δ2 + 8aa. An additional Sgv1 variant was engineered to contain an in-frame C-terminal Myc tag (EQKLISEEDL), which is labeled Sgv1-Myc. b) Unlike the high copy suppressor, *SUP3*, which suppresses the caffeine-sensitive growth of H3K36R-mutant cells, the WT *SGV1* clone does not suppress the cells; however, an *SGV1* clone containing the C-terminal addition of either the suppressor screen-associated changes (Δ2 + 8 amino acids), *sgv1Δ2 + 8aa*, or a Myc tag, *SGV1-Myc*, suppresses growth on caffeine. c) While the control *SGV1-Myc* suppresses the caffeine-sensitive growth of H3K36-mutant cells, *SGV1-Myc* containing either the catalytic amino acid substitution E107Q or D213A (Keogh *et al.* 2003) does not suppress the caffeine-sensitive growth defect of H3K36R-mutant cells.

The Sgv1 protein (also termed Bur1) is an evolutionarily conserved cyclin-dependent kinase (Irie *et al.* 1991), which is closely related to human CDK9 (Malumbres 2014; Chun *et al.* 2019), that is required for efficient transcription elongation by RNA polymerase II (RNAPII) (Keogh *et al.* 2003). Importantly, Sgv1/Bur1 phosphorylates the Rbp1 linker region between the RNAPII body and C-terminal domain (CTD), facilitating recruitment of the elongation factor Spt6 (Chun *et al.* 2019), and phosphorylates the C-terminal region of elongation factor Spt5 (Zhou *et al.* 2009). Consistent with this function, the Sgv1 protein contains a protein kinase domain (Fig. 5a). Although *SGV1* was the sole intact gene present on the *SUP3* suppressor, the C-terminal end of the Sgv1 open reading frame is truncated by 2 amino acids (loss of LY) with a predicted addition of 8 amino acids (PRVPSSNS) due to translation into the multiple cloning site of the vector (Fig. 5a). Thus, we cloned *SGV1* and tested for suppression of the caffeine-sensitive growth of H3K36R-mutant cells. Surprisingly, the WT *SGV1* clone does not suppress this growth defect (Fig. 5b). We then considered the possibility that the C-terminal loss of either 2 amino acids (Δ2) or addition of the 8 amino acids (+8AA) could confer suppression through a dominant mechanism. We thus reengineered a clone to independently produce the same Sgv1 variant (Fig. 5a, termed sgv1Δ2 + 8 amino acids or sgv1Δ2 + 8aa) as the one identified in the high copy suppressor screen. As shown in Fig. 5b, this Sgv1 variant suppresses the H3K36R growth defect on caffeine. We then considered the possibility that any C-terminal extension of Sgv1 is sufficient to mediate this suppression, so we appended a Myc tag (EQKLISEEDL) to the C-terminus of the WT *SGV1* open reading frame (Fig. 5a termed Sgv1-Myc). Interestingly, this C-terminally Myc-tagged Sgv1 protein also suppresses the caffeine-sensitive growth of H3K36R-mutant cells (Fig. 5b). Importantly, all the engineered clones contain the endogenous *SGV1* 3′ UTR so differences should not be due to changes in 3′-UTR-mediated regulation.

Having found that addition of Myc tag to the C-terminus of Sgv1 creates a functional suppressor of the H3K36 growth defect on caffeine (Fig. 5b), we next tested whether the catalytic activity of Sgv1 is required for this suppression. In this Sgv1-Myc variant, we generated 2 previously characterized mutants that impair the catalytic function of Sgv1/Bur1—*sgv1-E107Q* and *sgv1-D213A* (Keogh *et al.* 2003). As shown in Fig. 5c, when either of these changes that impair the catalytic activity of Sgv1 are introduced into Sgv1-Myc, no growth suppression is observed, arguing that the catalytic function of Sgv1 is required to suppress the caffeine-sensitive growth of H3K36R-mutant cells. In support of this conclusion, introduction of the E107Q or D213A catalytic mutations into the Sgv1-containing *SUP3* suppressor also impairs its ability to suppress H3K36R growth on caffeine (Supplementary Fig. 3). Thus, both the catalytic function and a change to the C-terminus of Sgv1 are required to suppress the growth of the histone-mutant cells.

**Fig. 6.**
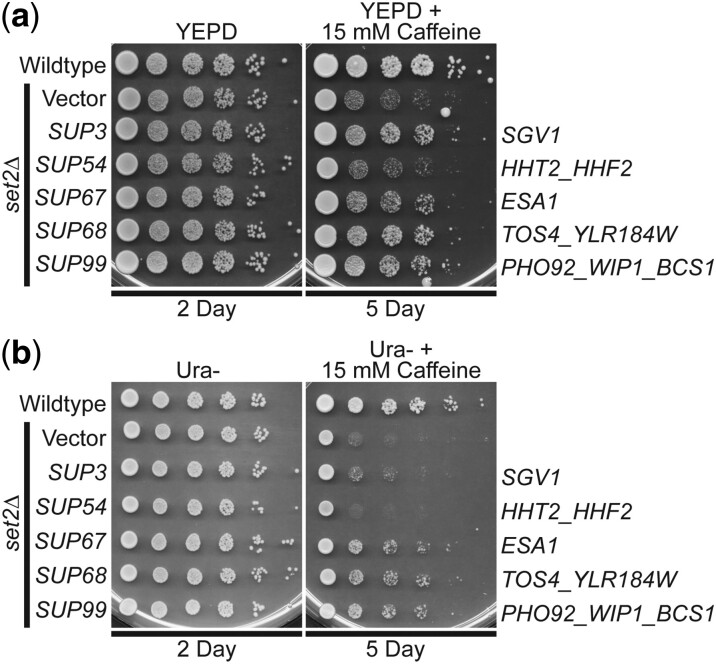
The identified genomic suppressors of H3K36R/M histone mutants, containing *SGV1*, *ESA1*, *TOS4*, and *PHO92*, suppress the caffeine-sensitive growth of *set2Δ* H3K36 histone methyltransferase-mutant cells on either a) YEPD plates with 15 mM caffeine or b) Ura− plates with 15 mM caffeine. The *set2Δ*-mutant cells containing *SUP3* (*SGV1*), *SUP67* (*ESA1*), *SUP68* (*TOS4*), and *SUP99* (*PHO92*) suppressor plasmids show improved growth on a caffeine plate compared to cells containing vector alone, but cells containing *SUP54* (*HHT2*) do not improve growth. The gene(s) encoded on the suppressor clones are indicated to right. WT cells transformed with vector and *set2Δ-*mutant cells transformed with vector, *SUP3*, *SUP54*, *SUP67*, *SUP68*, or *SUP99* plasmid were serially diluted and spotted onto a) a control YEPD plate and a YEPD plate containing 15 mM caffeine or b) a control Ura− plate and a Ura− plate containing 15 mM caffeine and grown at 30°C for indicated days.

Given that these suppressors were identified in a screen for genes that could suppress growth defects of either H3K36M- or H3K36R-mutant cells on plates containing caffeine, the mechanism of suppression is likely linked to PTM of this critical Lys36 residue in histone H3. To test this idea, we examined whether the identified genomic suppressors can suppress the caffeine-sensitive growth of budding yeast cells lacking the H3K36 histone methyltransferase, Set2 (Strahl *et al.* 2002; McDaniel *et al.* 2017). Four of the 5 identified genomic suppressors—*SUP3* (*SGV1*), *SUP67* (*ESA1*), *SUP68* (*TOS4*), and *SUP99* (*PHO92*)—can suppress the caffeine-sensitive growth of *set2Δ* cells (Fig. 6a). As expected suppressor *SUP54* (*HHT2*) does not suppress the *set2Δ* caffeine-sensitive growth defect (Fig. 6). This result is expected because an increase in the dose of histone H3 gene in the absence of a functional histone methyltransferase would not be expected to overcome the growth defect caused by loss of the methyltransferase and the accompanying methylation of H3K36. To directly compare these results to a previous study that analyzed high copy suppressors of *set2Δ* (McDaniel *et al.* 2017), the suppressors were also analyzed on Ura− plates with caffeine (Fig. 6b). These data functionally link these suppressors to proper methylation of H3K36.

## Discussion

Here, we leverage the eukaryotic *S. cerevisiae* model system to unmask the mechanism(s) of action for known human oncohistone mutations. Budding yeast is an attractive model system for these experiments as histones are highly conserved, the budding yeast genome is streamlined with only 2 H3 genes instead of the 15 H3 genes present in the mammalian genome, and essential biological pathways that contribute to growth and metabolism are evolutionarily conserved. Because the *S. cerevisiae* genome contains only 2 copies of histone H3, *HHT1* and *HHT2*, this model enables robust genetic screening to: (1) identify pathways and biological processes that are altered to support oncogenicity in mammals; and (2) apply this information to develop and design rational therapeutics for the treatment of oncohistone-driven cancers.

Here we focus on the defined oncohistones H3K36M, H3G34W/L/R/V, and the histone missense mutation H3K36R. While not oncogenic, H3K36R serves as a model to explore how a conservative amino acid change at this position alters function (McDaniel *et al.* 2017). Initial experiments compare the growth of yeast cells that express these histone H3 variants as the sole copy of H3. The data reveal distinct growth phenotypes between H3K36 and H3G34 mutants (Fig. 2), suggesting that while missense mutations that alter H3G34 reduce neighboring H3K36 methylation (Fig. 1e), the functional dynamics are more complex. Furthermore, while H3K36-mutant cells show growth defects on plates with caffeine, they show no growth defect on plates containing HU. In contrast, the H3G34-mutant cells all show growth defects in the presence of caffeine. Even cells with a significant decrease in the level of H3K36me3 such as H3G34V (∼25% of control H3K36me3) show no detectable growth defect on plates containing caffeine. These results align with recent studies that performed a similar analysis using *S. pombe* (Yadav *et al.* 2017; Lowe *et al.* 2021). The differences observed between oncohistone variants in yeast are also congruent with the different types of cancers linked to somatic mutations that alter H3K36 or H3G34 (Wan *et al.* 2018). Combined, these data suggest that additional analyses are required to uncover precisely how different amino acid changes within histones alter biological pathways to drive oncogenesis.

To define the biological pathways altered by different histone mutations, we exploited yeast genetics to perform a high copy suppressor screen. In this screen, we identified 4 candidate suppressors—the lysine acetyltransferase, Esa1, the gene expression regulator, Tos4, the m^6^A RNA binding protein Pho92, and the cyclin-dependent kinase, Sgv1/Bur1—that rescue the caffeine-associated growth defects that are exhibited by histone H3K36 missense mutations, which are known oncogenic drivers in cancer. The suppressors identified primarily suppress growth on caffeine with minimal effects on the other drugs that impair growth of the different histone models, suggesting that different biological pathways, which are impacted by the oncohistone missense mutations, can be defined with this genetic approach. Caffeine induces a stress response that depends on the Tor pathway (Kuranda *et al.* 2006), which is evolutionarily conserved as the mTOR pathway in humans (Tatebe and Shiozaki 2017). Identifying genes involved in suppressing growth defects associated with Tor pathway-induced stress is biologically relevant to human cancers, as the mammalian PI3K/AKT/mTOR signaling pathway mediates essential biological processes that are frequently deregulated in cancer: cell growth, survival, proliferation, and metabolism (Saxton and Sabatini 2017).

The high copy suppressor screen identified the histone H4/H2A lysine acetyltransferase Esa1 as a suppressor of caffeine-sensitive growth of H3K36M and H3K36R histone-mutant cells (Figs. 2 and 3). The lysine acetyltransferase activity of Esa1 is required for the suppression as 2 independent catalytically inactive mutants of Esa1, Esa1 C304S and Esa1 E338Q (Yan *et al.* 2000; Decker *et al.* 2008), do not suppress the caffeine-sensitive growth of H3K36R-mutant cells. Interestingly, di- and trimethylation of histone H3K36 stimulates the interaction of the NuA4 lysine acetylation complex, which contains the Esa1 catalytic subunit, with nucleosomes and NuA4 acetylation of lysine residues in the histone H4 tail has also been observed to stimulate the interaction of the SAGA lysine acetylation complex with nucleosomes, enhancing acetylation of histone H3 (Ginsburg *et al.* 2014). Overexpression of Esa1 in H3K36R-mutant cells could therefore increase acetylation of histone H4 to enhance SAGA recruitment to nucleosomes to restore optimal acetylation of histone H3.

Crosstalk between H4 and/or H2A acetylation and H3K36 methylation could potentially impact cell growth or other pro-oncogenic properties that are abrogated in the absence of Esa1 enzymatic activity. However, the human homologue of Esa1, KAT5/TIP60, has additional nonhistone substrates (Sapountzi and Côté 2011) that may impact biological function in high copy suppressor assays such as those employed here. TIP60 is involved in DNA repair through the acetylation of nonhistone proteins such as the ATM kinase (Sun *et al.* 2007). Future experiments could include determining whether Esa1 suppresses growth in the presence of DNA damaging agents such as HU, and whether this is dependent on Esa1 acetyltransferase activity. Ultimately, defining the relevant targets of Esa1 that confer growth suppression will lend insight into the biological pathways altered by oncogenic changes at H3K36.

The high copy screen also identified the gene expression regulator Tos4 as a potent suppressor of the caffeine-sensitive growth of H3K36-mutant cells. Tos4 binds to the yeast HDAC complexes Rpd3L and Set3 via a FHA domain (Fig. 4a) (Hofmann and Bucher 1995; Bastos de Oliveira *et al.* 2012; Cooke *et al.* 2021), which ultimately leads to histone deacetylation. However, amino acids substitutions that interfere with Rpd3L/Set3 complex binding to the FHA domain of Tos4 did not affect Tos4-mediated H3K36-mutant growth suppression (Fig. 4b), suggesting that Rpd3L/Set3-independent functions of Tos4 mediate suppression. This model is supported by the fact that we did not independently identify Rpd3L or Set3 complex genes as growth suppressors in our initial high copy suppressor screen. Pho92, an RNA “reader” protein that recognizes and binds m^6^A-containing RNA (Shi *et al.* 2021) is another suppressor identified in this screen. A previous screen identified *PHO85* as a high copy suppressor of caffeine-sensitive growth of *set2Δ* cells and explored the link to the nutrient sensing pathway (McDaniel *et al.* 2017). In this pathway, Pho92 regulates the stability of the *PHO4* transcript (Kang *et al.* 2014), which encodes a helix-loop-helix transcription factor that activates transcription in response to limiting phosphate (Ogawa and Oshima 1990). However, amino acid substitutions within the reader domain that render Pho92 incapable of binding m^6^A RNA do not alter Pho92-mediated growth suppression of the H3K36R-mutant growth, suggesting the mechanism(s) by which Pho92 mediates suppression is independent of recognizing and/or binding m^6^A RNA. Collectively, more research is needed to mechanistically define how some suppressors such as Tos4 and Pho92 suppress growth defects of H3K36-mutant cells.

The high copy suppressor screen also identified a clone containing nearly all of the *S. cerevisiae* kinase Sgv1/Bur1 open reading frame, a homologue of human CDK9 (Fig. 5b). Previous studies have defined functional links between Sgv1/Bur1 and H3K36 methylation, demonstrating that a functional Sgv1/Bur1 protein is required for normal H3K36 methylation (Chu *et al.* 2006). While the slightly truncated *SGV1* genomic suppressor identified (*SUP3*) suppresses the caffeine-sensitive growth of H3K36R-mutant cells, the full-length *SGV1* clone does not. The genomic *SGV1* clone that was identified as a suppressor encodes a truncated Sgv1 protein, lacking the 2 C-terminal amino acids (LY) and fused to an additional 8 amino acids (PRVPSSNS) prior to reaching the next natural stop codon. As appending a C-terminal Myc tag with a very distinct sequence (EQKLISEEDL) to Sgv1 also suppresses the caffeine-sensitive growth of H3K36-mutant cells (Fig. 5, b and c), the nature of the change to the C-terminus of Sgv1 is clearly not important. Furthermore, both the modified C-terminal end of the protein and the catalytic activity are required for suppression, suggesting a dominant function that relies on Sgv1 kinase activity. The CTD of *S. cerevisiae* Sgv1 is not evolutionarily conserved in mammalian CDK9 orthologs, but the CTD of Sgv1 is implicated in interaction with the RPA protein to ensure genome stability (Clausing *et al.* 2010). In addition, the CTD of Sgv1 does share identity with the CTD of *S. pombe* Cdk9, which interacts with the mRNA capping/RNA 5′-triphophatase enzyme, Pct1 (Cet1 in *S. cerevisiae*) (Takagi *et al.* 2002; Pei and Shuman 2003). A previous screen found that overexpression of Sgv1 induced aberrant budding (Sopko *et al.* 2006), so it will be interesting to test the variants of Sgv1 created here to assess whether they recapitulate this effect observed for WT Sgv1. Collectively, these data suggest that we may have identified a regulatory role for the C-terminal end of Sgv1 that depends on active kinase activity. We also note that the *SGV1* suppressor (*SUP3*) shows more robust suppression on YEPD plates with caffeine as compared to Ura− plates with caffeine (compare Fig. 6, a and b). Clearly further studies will be required to understand both the function of the C-terminus of Sgv1 and the interplay between Sgv1 and H3K36 oncohistone models.

The high copy suppressor screen performed here provides a streamlined and rapid approach to identify genetic vulnerabilities that may be therapeutically actionable. Two of the suppressors identified, Esa1 and Sgv1, encode enzymes that have been targeted in the corresponding pathways in human cells as potential therapies. Expression of KAT5/TIP60 lysine acetyl transferase, the human ortholog of Esa1, is deregulated in prostate and other cancers (Shiota *et al.* 2010). The preclinical TIP60 acetyltransferase inhibitors Nu9056 and TH1834 reduce DNA-damage induced ATM phosphorylation (Gao *et al.* 2014). Thus, TIP60 inhibition may be a viable therapeutic strategy for cancers dependent on TIP60 enzymatic activity. This experimental approach in budding yeast provides the first evidence that H3K36-mutant tumors may be candidates for TIP60 inhibition, but subsequent experimentation in H3K36-mutant human cancer cell lines is required to test this hypothesis. The human homologue of Sgv1, CDK9 (Chun *et al.* 2019), binds to Cyclin T to form the positive transcription elongation factor complex (P-TEFb); CDK9 kinase activity is thus critical for RNA-Pol II-directed transcription (Bacon and D’Orso 2019). CDK9 is deregulated in hematological malignancies and other cancer types, prompting the development of CDK9 inhibitors (Mandal *et al.* 2021). However, understanding the mechanism(s) by which CDK9 inhibition contributes to anti-cancer effects is still under investigation. The CDK9 inhibitor AZD4573 is the first CDK9-selective inhibitor (Cidado *et al.* 2020) and has entered clinical trials for hematological cancers. The anti-tumorigenic activity of CDK9 inhibition is thought to be due to the depletion of the anti-apoptotic protein MCL-1 (Dey *et al.* 2017). More recently, H3K27M oncohistone-expressing diffuse midline gliomas (DMGs) have been shown to deregulate the expression of AFF4, a scaffolding protein involved in transcriptional elongation, which CDK9/CyclinT regulates (Dahl *et al.* 2020). These preclinical studies have prompted the clinical testing of AZD4573 in hematologic malignancies (Rule *et al.* 2018; Barlaam *et al.* 2020) and suggest that H3K27M-mutant tumors that are characterized by AFF4 upregulation are candidates for clinical CDK9 inhibition (Barlaam *et al.* 2020). While through a divergent mechanism, our data suggest that CDK9 inhibition may be beneficial for H3K36-mutant head and neck cancers and chondroblastomas, although further research is necessary to test this hypothesis.

Taken together, the results from this suppressor screen exploiting the budding yeast model both define novel cellular pathways that could be altered by missense mutations in histones that drive oncogenesis and uncover links to potential therapeutic avenues. Future studies will help to define the mechanisms by which the suppressors rescue the growth of the oncohistone yeast models, which may further define how these pathways could be targeted for therapeutic approaches.

## Data availability

Strains and plasmids are available upon request. The authors affirm that all data necessary for confirming the conclusions of the article are present within the article, figures, and tables.

*Supplemental material* is available at *G3* online.

## Supplementary Material

jkac120_Supplementary_Data
