# Supplementary material for: A Saccharomyces cerevisiae model and screen to define the functional consequences of oncogenic histone missense mutations
Source: G3 (Bethesda). 2022 May 14;12(7):jkac120. doi: 10.1093/g3journal/jkac120 (PMC9258546; doi:10.1093/g3journal/jkac120)
Supplement: jkac120_Supplementary_Data [file jkac120_supplementary_data.pdf]

Supplemental Table 1

Strain/Plasmid	Description	Source
Wildtype (yAAD1253)	*MATα, ura3-52, leu2Δ1, his3Δ200, lys2-128δ*	(DUINA AND TURKAL 2017)
hht2Δ (yAAD165)	*MATa, ura3-52, leu2Δ1, his3Δ200, trp1Δ63, lys2-128δ, hht2Δ::URA3:TRP1*	(DUINA AND TURKAL 2017)
hht1Δ (ACY2818)	*MATα, ura3-52, leu2Δ1 his3Δ200, lys2-128δ, hht1Δ::kanMX*	This Study
hht2-K36R (ACY2816)	*MATa, ura3-52, leu2Δ1, his3Δ200, trp1Δ63, lys2-128δ, hht2-K36R*	This Study
hht2-K36R hht1Δ (ACY2821)	*MATa, ura3-52, leu2Δ1, his3Δ200, trp1Δ63, lys2-128δ, hht2-K36R, hht1Δ::kanMX*	This Study
hht2-K36M (ACY2830)	*MATa, ura3-52, leu2Δ1, his3Δ200, trp1Δ63, lys2-128δ, hht2-K36M*	This Study
hht2-K36M hht1Δ (ACY2822)	*MATa, ura3-52, leu2Δ1, his3Δ200, trp1Δ63, lys2-128δ, hht2-K36M, hht1Δ::kanMX*	This Study
hht2-G34W (ACY2823)	*MATa, ura3-52, leu2Δ1, his3Δ200, trp1Δ63, lys2-128δ, hht2-G34W*	This Study
hht2-G34W hht1Δ (ACY2825)	*MATa, ura3-52, leu2Δ1, his3Δ200, trp1Δ63, lys2-128δ, hht2-G34W, hht1Δ::kanMX*	This Study
hht2-G34L (ACY2831)	*MATa, ura3-52, leu2Δ1, his3Δ200, trp1Δ63, lys2-128δ, hht2-G34L*	This Study
hht2-G34L hht1Δ (ACY2833)	*MATa, ura3-52, leu2Δ1, his3Δ200, trp1Δ63, lys2-128δ, hht2-G34L, hht1Δ::kanMX*	This Study
hht2-G34R (ACY2838)	*MATa, ura3-52, leu2Δ1, his3Δ200, trp1Δ63, lys2-128δ, hht2-G34R*	This Study
hht2-G34R hht1Δ (ACY2840)	*MATa, ura3-52, leu2Δ1, his3Δ200, trp1Δ63, lys2-128δ, hht2-G34R, hht1Δ::kanMX*	This Study
hht2-G34V (ACY2841)	*MATa, ura3-52, leu2Δ1, his3Δ200, trp1Δ63, lys2-128δ, hht2-G34V*	This Study
hht2-G34V hht1Δ (ACY2846)	*MATa, ura3-52, leu2Δ1, his3Δ200, trp1Δ63, lys2-128δ, hht2-G34V, hht1Δ::kanMX*	This Study
set2Δ (ACY2851)	*MATα, ura3-52, leu2Δ1, his3Δ200, lys2-128δ, set2Δ::kanMX*	This Study
YEp352 (pAC29)	*URA3, 2μ, amp^R*	(HILL *et al.* 1986)
SUP3 (pAC4132)	*SGV1, URA3, 2μ, amp^R*	This Study
SUP54 (pAC4145)	*HHT2, HHF2, URA3, 2μ, amp^R*	This Study
SUP67 (pAC4149)	*ESA1, URA3, 2μ, amp^R*	This Study
SUP68 (pAC4150)	*TOS4, YLR184W, URA3, 2μ, amp^R*	This Study
SUP99 (pAC4160)	*PHO92, WIP1, BCS1, URA3, 2μ, amp^R*	This Study
HHF2 (pAC4199)	*HHF2, URA3, 2μ, amp^R*	This Study
HHT2 (pAC4201)	*HHT2, URA3, 2μ, amp^R*	This Study
HHT1 (pAC4200)	*HHT1, URA3, 2μ, amp^R*	This Study
ESA1 (pAC4190)	*ESA1, URA3, 2μ, amp^R*	This Study
esa1-C304S (pAC4191)	*esa1-C304S, URA3, 2μ, amp^R*	This Study
esa1-E338Q (pAC4192)	*esa1-E338Q, URA3, 2μ, amp^R*	This Study
TOS4 (pAC4196)	*TOS4, URA3, 2μ, amp^R*	This Study
tos4-R122A-N161A (pAC4205)	*tos4-R122A-N161A, URA3, 2μ, amp^R*	This Study
PHO92 (pAC4193)	*PHO92, URA3, 2μ, amp^R*	This Study
pho92-W177A (pAC4194)	*pho92-W177A, URA3, 2μ, amp^R*	This Study
pho92-W231A (pAC4195)	*pho92-W231A, URA3, 2μ, amp^R*	This Study
SGV1 (pAC4187)	*SGV1, URA3, 2μ, amp^R*	This Study
sgv1-E107Q (pAC4188)	*sgv1-E107Q, URA3; 2μ, amp^R*	This Study
sgv1-D213A (pAC4189)	*sgv1-D213A, URA3; 2μ, amp^R*	This Study
sgv1-Δ2+8aa (pAC4208)	sgv1-Δ2+8aa, URA3, 2μ, *amp^R*	This Study
SGV1-Myc (pAC4209)	*SGV1-Myc, URA3, 2μ, amp^R*	This Study
sgv1-E107Q-Myc (pAC4210)	*sgv1-E107Q-Myc, URA3, 2μ, amp^R*	This Study
sgv1-D213A-Myc (pAC4211)	*sgv1-D213A-Myc, URA3, 2μ, amp^R*	This Study
SUP3-E107Q (pAC4212)	*SUP3-E107Q, URA3, 2μ, amp^R*	This Study
SUP3-D213A (pAC4213)	*SUP3-D213A, URA3, 2μ, amp^R*	This Study

Supplemental Figure 1

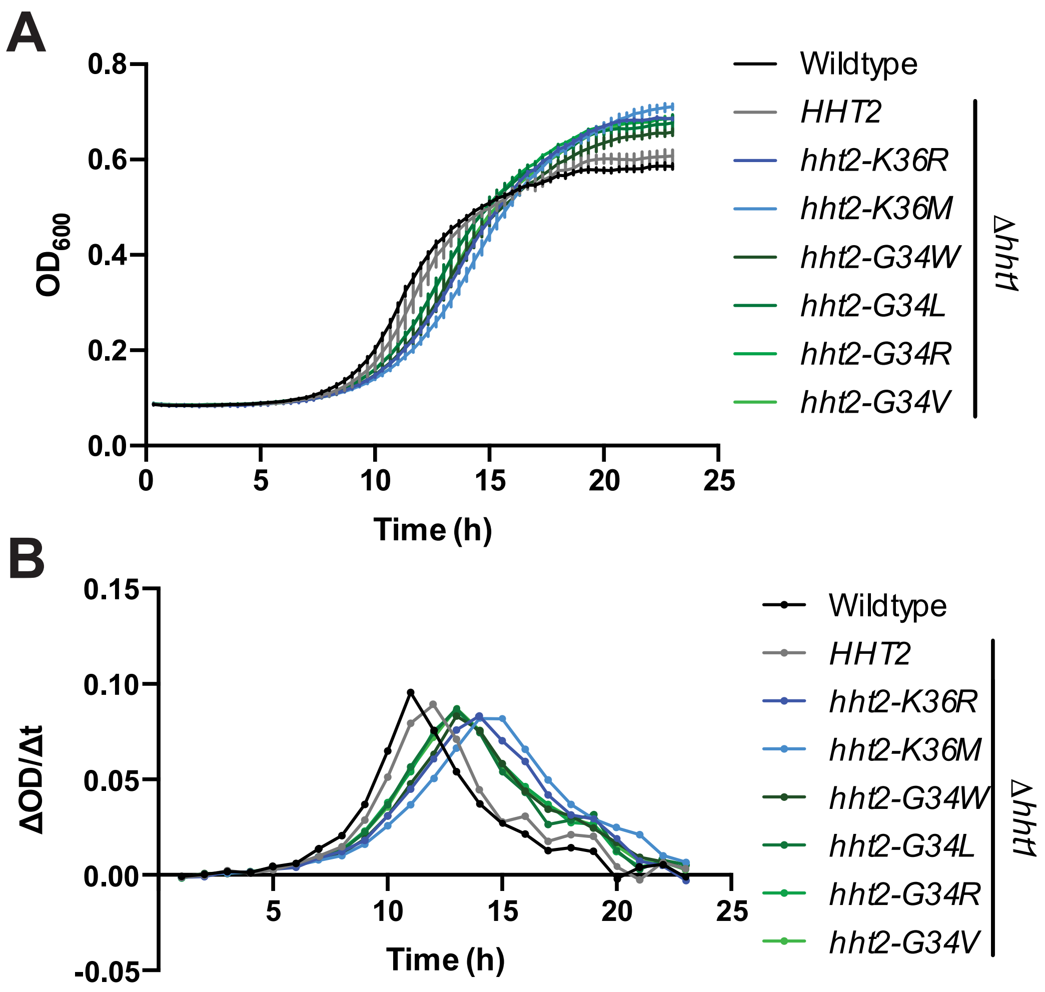

Figure S1. (A) Control Wildtype and *HHT2 hht1*Δ cells (black), H3K36 mutant cells (*hht2-K36R/M hht1*Δ) (blue), or H3G34 mutant cells (*hht2-G34W/L/R/V hht1*Δ) (green) were grown in YEPD liquid media and growth was assessed my measuring OD600 every 20 minutes in a plate reader for 24 hours. (B) The area under the growth curve was obtained for each of the mutants analyzed, revealing that the histone mutant cells achieve a higher biomass than either control.

Supplemental Figure 2

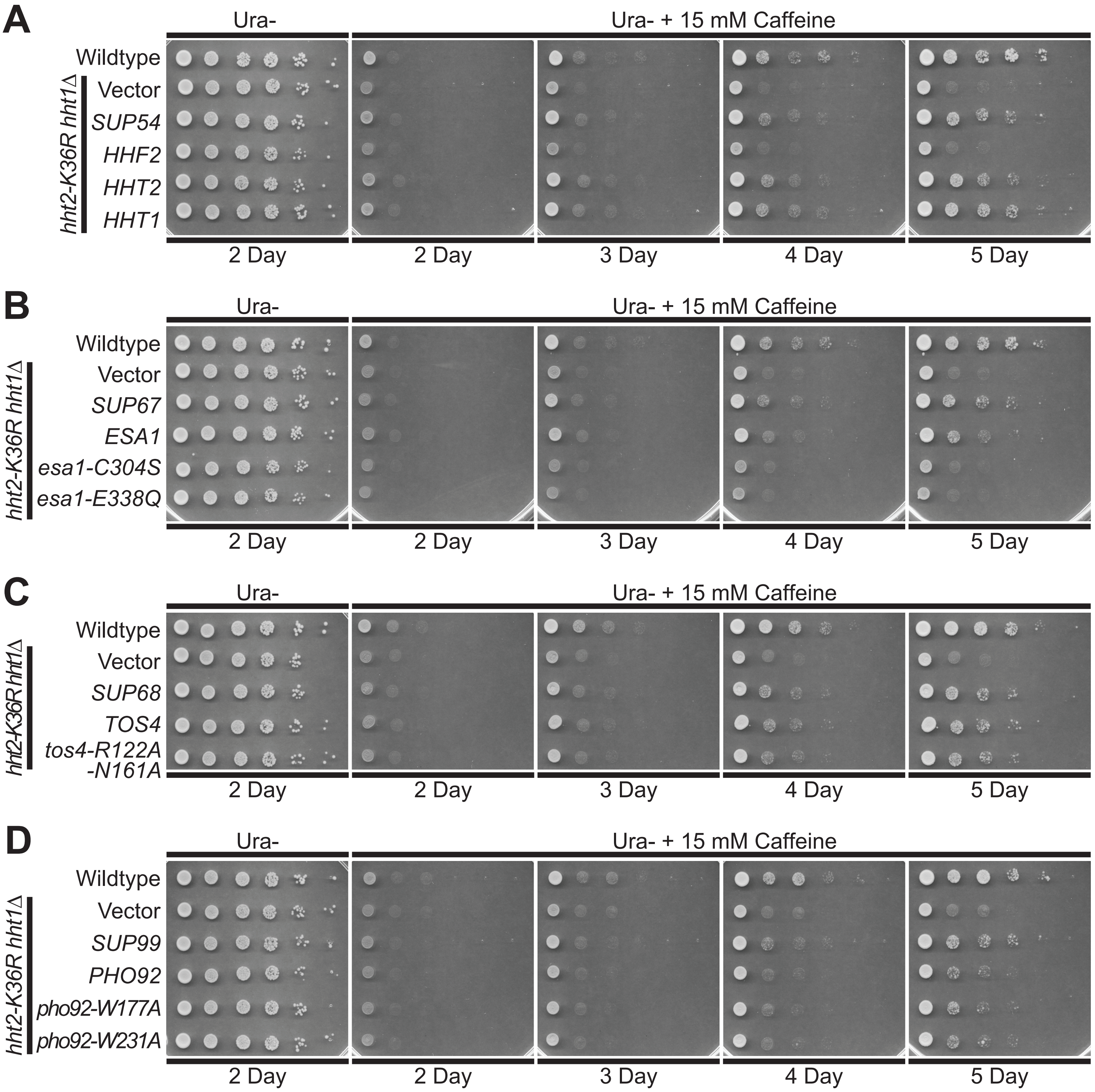

Figure S2. High copy suppressors identified suppress caffeine sensitive growth of H3K36R cells on Ura$^-$ plates. For the following high copy suppressors: (A) *HHT2*, (B) *ESA1*, (C) *TOS4*, and (D) *PHO92* serial dilution growth assays are show for the original *SUP* clone isolated, the subcloned suppressor, and any variants tested on Ura$^-$ plates that contain 15 mM caffeine. Results on plates with caffeine are shown for Day 2 to Day 5.

Supplemental Figure 3

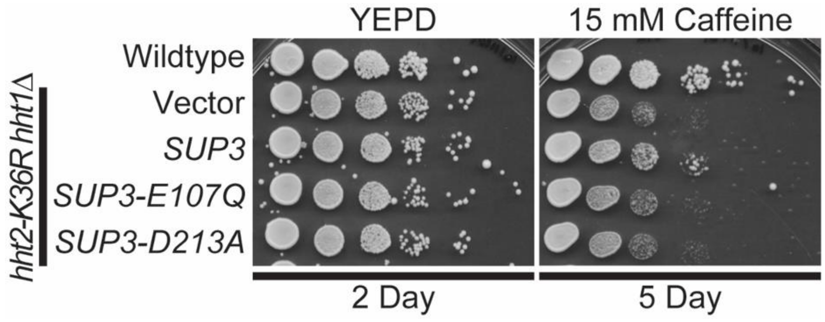

Figure S3. Both the catalytic activity of Sgv1 and a C-terminal extension are required for Sgv1-mediated suppression of H3K36R caffeine sensitive growth. Using the original suppressor clone, termed *SUP3*, either of two Sgv1 amino acid substitutions that impair Sgv1 catalytic activity (E107Q or D213A) (KEOGH *et al.* 2003) engineered into the original suppressor clone, *SUP3*, identified abrogate suppression.

Supplemental References

Duina, A. A., and C. E. Turkal, 2017 Targeted in Situ Mutagenesis of Histone Genes in Budding Yeast. J Vis Exp**:** 55263.

Hill, J. E., A. M. Myers, T. J. Koerner and A. Tzagoloff, 1986 Yeast/E. coli shuttle vectors with multiple unique restriction sites. Yeast 2**:** 163-167.

Keogh, M. C., V. Podolny and S. Buratowski, 2003 Bur1 kinase is required for efficient transcription elongation by RNA polymerase II. Mol Cell Biol 23**:** 7005-7018.
